# Direct Hydroxylation of Benzene with Hydrogen Peroxide Using Fe Complexes Encapsulated into Mesoporous Y-Type Zeolite

**DOI:** 10.3390/molecules27206852

**Published:** 2022-10-13

**Authors:** Syuhei Yamaguchi, Yuito Ishida, Hitomu Koga, Hidenori Yahiro

**Affiliations:** Department of Materials Science and Biotechnology, Graduate School of Science and Engineering, Ehime University, 3 Bunkyo-cho, Matsuyama 791-8577, Japan

**Keywords:** Fe complex, benzene oxidation, zeolite, mesopore

## Abstract

Mesoporous Y-type zeolite (MYZ) was prepared by an acid and base treatment of commercial Y-type zeolite (YZ). The mesopore volume of MYZ was six times higher than that of YZ. [Fe(terpy)_2_]^2+^ complexes encapsulated into MYZ and YZ with different Fe contents (Fe(X)L-MYZ and Fe(X)L-YZ; X is the amount of Fe) were prepared and characterized. The oxidation of benzene with H_2_O_2_ using Fe(X)L-MYZ and Fe(X)L-YZ catalysts was carried out; phenol was selectively produced with all Fe-containing zeolite catalysts. As a result, the oxidation activity of benzene increased with increasing iron complex content in the Fe(X)L-MYZ and Fe(X)L-YZ catalysts. The oxidation activity of benzene using Fe(X)L-MYZ catalyst was higher than that using Fe(X)L-YZ. Furthermore, adding mesopores increased the catalytic activity of the iron complex as the iron complex content increased.

## 1. Introduction

The direct catalytic hydroxylation of inert C–H bonds in hydrocarbons under mild conditions is a major challenge in synthetic chemistry in the chemical industry or in academic research [1,2,3,4]. In particular, the catalytic hydroxylation of benzene to phenol using environmentally friendly oxidants, such as hydrogen peroxide (H_2_O_2_) [5,6,7,8,9,10,11,12], molecular oxygen (O_2_) in combination with reducing agents [13,14,15,16,17,18], and H_2_O with electrochemical [19] or photochemical reaction systems [20,21] has attracted considerable attention. One of the most attractive areas in catalysis is the development of inorganic–organic hybrid materials that are active for oxidation reactions [12,22,23,24,25,26,27,28,29,30,31,32,33,34,35,36]. Many researchers have reported the oxidation of organic substrates (benzene [12,24,32,33,34], phenol [25,26], cyclohexane [23,27,33], cyclohexene [23,28,30,31,33], and sulfides [34]) with H_2_O_2_ as an oxidant using transition metal complexes encapsulated in Y-type zeolite.

A previous study reported the catalytic activity of Fe-bipyridine complexes encapsulated into Y-type zeolites ([Fe(bpy)_3_]^2+^@Na-Y), which can catalyze the oxidation of benzene and cyclohexene with H_2_O_2_ in CH_3_CN and H_2_O as solvents [30,31,32,33]. The maximum catalytic activity of [Fe(bpy)_3_]^2+^@Na-Y for the oxidation of benzene with H_2_O_2_ was achieved when the volume ratio of the solvents (CH_3_CN and H_2_O) was 1:1 [32]. [Fe(terpy)_2_]^2+^@Na-Y and [Fe(phen)_3_]^2+^@Na-Y (terpy = 2,2′;6′,2″-terpyridine and phen = 1,10-phenanthroline) exhibited a higher catalytic activity than [Fe(bpy)_3_]^2+^@Na-Y in the oxidation of benzene with H_2_O_2_ to phenol [33].

Substantial progress has been made in the synthesis, characterization, and catalytic exploitation of hierarchically structured variants of Faujasite (X, Y, and USY) zeolites [37,38,39]. Verboekend et al. [37,38] reported that mesoporous zeolite could be prepared from Y-type zeolite by acid and base treatments. The mesoporous zeolite prepared by acid–base treatment of Y-type zeolite exhibited higher catalytic activity for the alkylation of toluene with benzyl alcohol than untreated Y-type zeolite.

The mesoporous zeolite support for [Fe(terpy)_2_]^2+^@Na-Y was prepared by an acid–base treatment of Y-type zeolite to improve the catalytic activity for benzene oxidation. In this study, the catalytic activity for the oxidation of benzene with H_2_O_2_ was investigated using [Fe(terpy)_2_]^2+^ complexes encapsulated into mesoporous Y-type zeolite.

## 2. Results and Discussion

### 2.1. Preparation and Characterization of Mesoporous Y-Type Zeolite

**Figure 1:**
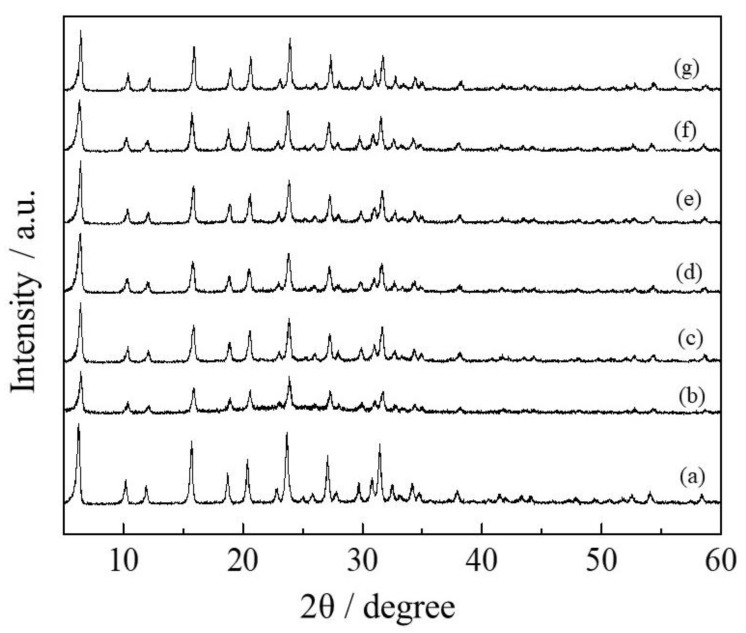
XRD patterns of MYZ-t and YZ samples. YZ (a), MYZ-t (t = 0 (b), 0.5 (c), 1.0 (d), 5.0 (e), 16 (f), and 24 (g)).

**Figure 2:**
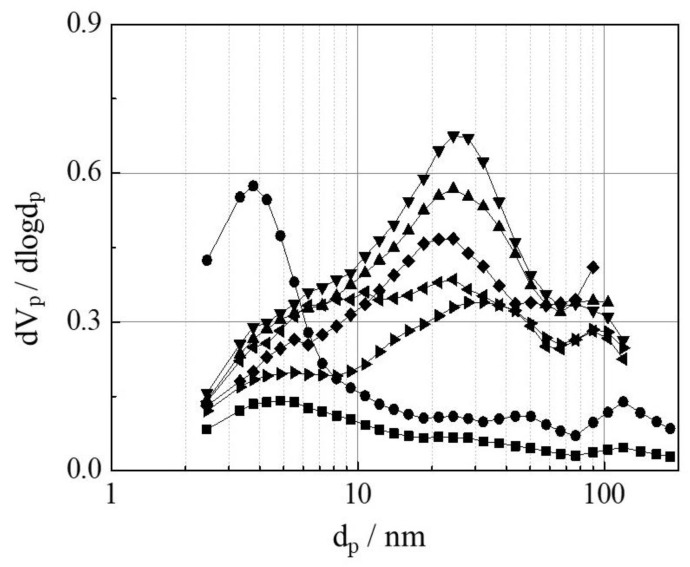
Pore distributions of MYZ-t and YZ samples. YZ (■), MYZ-t (t = 0 (●), 0.5 (▲), 1.0 (▼), 5.0 (◆), 16 (◄), and 24 (►)).

The mesoporous Y-type zeolites were prepared from commercial Y-type zeolite (YZ) by sequential acid (H_4_EDTA), base (NaOH), and acid (Na_2_H_2_EDTA) treatments. The acid- and base-treated zeolites to produce mesoporous Y-type zeolite were called MYZ-t (t = 0–24 h), where t is the base treatment time (hours (h)). Figure 1 shows X-ray diffraction (XRD) patterns of MYZ-t (t = 0–24 h) and original YZ. The XRD patterns of MYZ-t were similar to that of the original YZ, suggesting that the structure of Y-type zeolite had been maintained after the acid and base treatments. The N_2_ absorption–desorption isotherms of MYZ-t and YZ (Figure S1) provided IV and H4 type isotherms [40], suggesting that MYZ-t and YZ have cylinder-like pores. Table 1 lists the N_2_ absorption–desorption parameters of MYZ-t and YZ. The mesopore volumes (V_meso_) of MYZ-t were much larger than those of the original YZ. The mesopore volume of MYZ-t increased to t = 1.0 (base treatment = 1.0 h) and decreased with further increases in t. Both total pore volume (V_total_) and mean pore diameter (d_p_), and the mesopore-specific surface area (S_meso_) of MYZ-1.0 were also the largest among the samples tested. Figure 2 shows the pore distributions of MYZ-t and YZ. In the case of MYZ-0, approximately 4 nm pores formed. On the other hand, in the case of MYZ-t (t > 0), the number of pores less than 10 nm in size decreased with a concomitant increase in the number of 20–30 nm mesopores. The number of 20–30 nm mesopores of MYZ-t increased up to t = 1.0 and decreased with further increases in t. Thus, among MYZ-t, MYZ-1.0 was adopted as a typical mesoporous Y-type zeolite.

### 2.2. Preparation and Characterization of the Fe Complexes Encapsulated into the Zeolite

The mesoporous Y-type zeolite catalysts (Fe(X)L-MYZ-t) and untreated zeolite catalysts (Fe(X)L-YZ) with different contents of iron complexes were prepared using a method reported elsewhere [30,31,32,33]. The X calculated by inductively coupled plasma–atomic emission spectroscopy (ICP-AES) represents the weight percentage concentration (wt. %) of Fe in Fe(X)L-MYZ-t and Fe(X)L-YZ catalysts. The ICP-AES and CHN elemental analysis indicated that the Fe ion in MYZ-t and YZ was coordinated with two terpy (terpy/ Fe = 2) ligands, suggesting the formation of [Fe(terpy)_2_]^2+^ ions in the Fe(X)L-MYZ-t and Fe(X)L-YZ catalysts.

**Figure 3:**
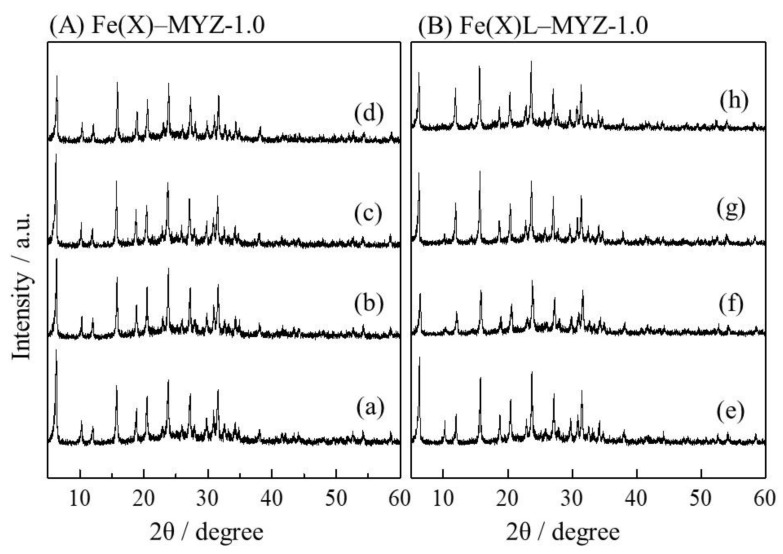
XRD patterns of Fe(X)-MYZ-1.0 (**A**) and Fe(X)L-MYZ-1.0 catalysts (**B**). X = 0.63 (a, e), 1.07 (b, f), 1.54 (c, g), and 2.11 (d, h).

**Figure 4:**
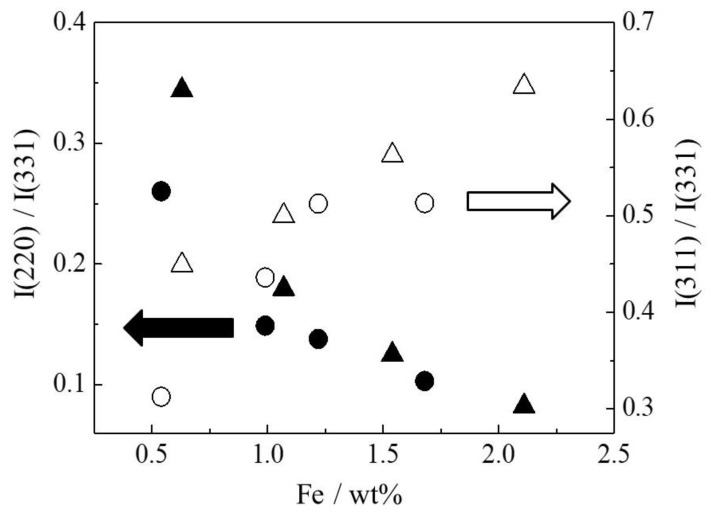
Relative intensity of (220) and (311) against (331) as a function of Fe content of Fe(X)L-MYZ-1.0 and Fe(X)L-YZ catalysts. (220) (2θ = 10°), (311) (2θ = 12°), and (331) (2θ = 16°). Fe(X)L-MYZ-1.0: (220) (▲), (311) (△); Fe(X)L-YZ: (220) (●), (311) (◯).

Figure 3 shows XRD patterns of the Fe(X)-MYZ-1.0 and Fe(X)L-MYZ-1.0 catalysts. The zeolite structure was maintained after the introduction of each metal complex. The empirically derived relationship between the relative peak intensities of the (220) and (311) reflection in the XRD pattern confirmed the formation of a large metal ion in a supercage of faujasite-type zeolite; the intensity of (220) for the zeolite containing large complexes is lower than that for the original zeolite Y, while the intensity of (311) for former is greater than that for the latter [12,31,33]. As shown in Figure 3, the intensities of the (220) reflection (2*θ* = 10°) for the Fe(X)L-MYZ-1.0 catalysts were lower than those for the corresponding Fe(X)-MYZ-1.0 samples, while the intensities of the (311) plane (2*θ* = 12°) in the former were greater than those of the latter. Similar changes in XRD peak intensity before and after ligand introduction were obtained for Fe(X)L-YZ (Figures S2 and S3) and FeL-MYZ-t (Figures S4 and S5) catalysts. The relative intensity of the (220) reflection to the (331) reflection (2*θ* = 16°) decreased with increasing Fe content in the Fe(X)L-MYZ-1.0 and Fe(X)L-YZ catalysts, while the (311) to (331) intensity ratio increased (Figure 4). These results provide clear evidence of the formation of metal complex ions within the supercage.

**Figure 5:**
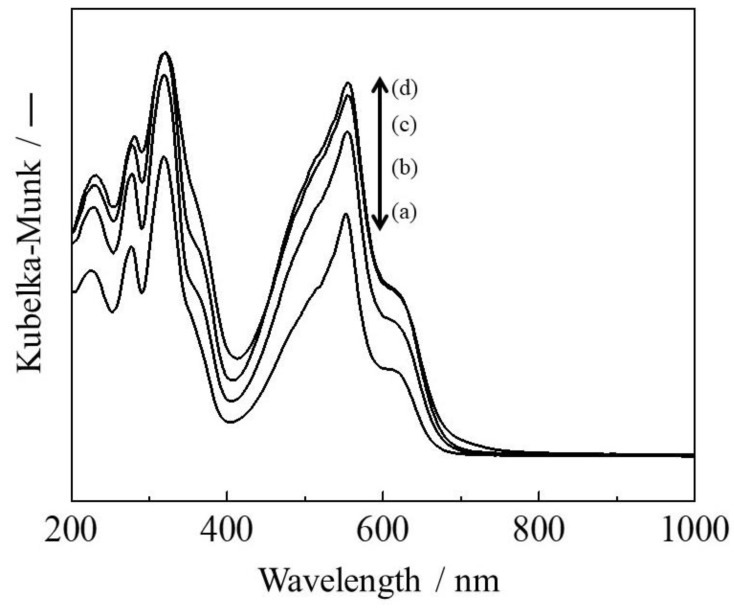
UV-vis spectra of Fe(X)L-MYZ-1.0 catalysts. X = 0.63 (a), 1.07 (b), 1.54 (c), and 2.11 (d).

Figure 5 presents the UV-vis diffuse reflectance spectra of the Fe(X)L-MYZ-1.0 catalysts. The catalysts showed no absorption in the ultraviolet to the visible region without the metal complex, Fe-YZ and YZ [33]. The absorption spectra of Fe(X)L-MYZ-1.0, Fe(X)L-YZ, and FeL-MYZ-t catalysts (Figure 5) produced two bands in the regions of 400–650 nm and 250–400 nm, which can be assigned to metal-to-ligand (d–π*) charge-transfer (MLCT) and a π–π* transition of terpy ligand of the [Fe(terpy)_2_]^2+^ complex, respectively, similar to that of [Fe(terpy)_2_](ClO_4_)_2_ [33]. The UV-vis spectral behaviors of Fe(X)L-YZ (Figure S6) and FeL-MYZ-t (Figure S7) catalysts were quite similar to those of Fe(X)L-MYZ-1.0 catalysts (Figure 5). The intensity of MLCT at 550 nm increased with increasing Fe content in the Fe(X)L-MYZ-1.0 and Fe(X)L-YZ catalysts (Figure 5 and Figure S6). These results suggest that the UV-vis spectral behavior of Fe complexes in zeolites is not affected by the presence or absence of mesopore. Elemental analysis, XRD, and UV-vis showed that the desired Fe complexes, [Fe(terpy)_2_]^2+^ were formed in the supercage of Y-type zeolite.

### 2.3. Oxidation of Benzene with Hydrogen Peroxide

**Figure 6:**
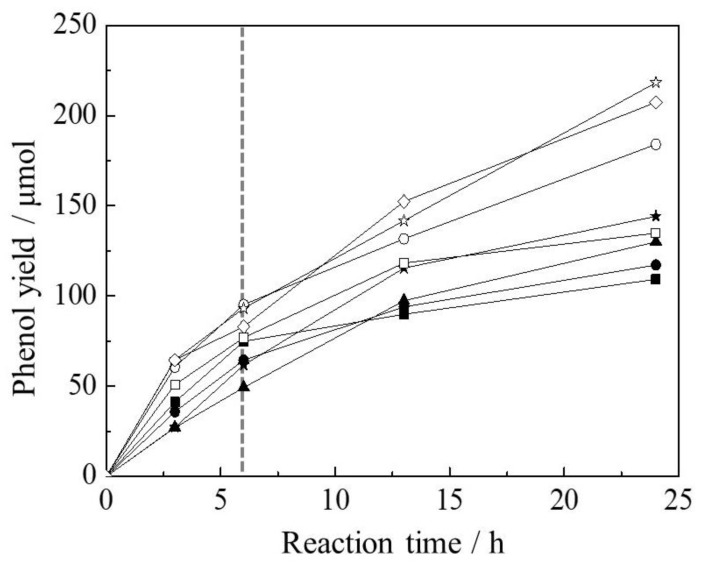
Time course for oxidation of benzene with H_2_O_2_ using Fe(X)L-MYZ-1.0 and Fe(X)L-YZ catalysts. Fe(X)L-MYZ-1.0; X = 0.63 (□), 1.07 (◯), 1.54 (✩), and 2.11 (◇). Fe(X)L-YZ; X = 0.54 (■), 0.99 (●), 1.22 (▲), and 1.68 (★). Reaction condition: Fe in catalysts (7.9 μmol), benzene (7.9 mmol), 30% aqueous H_2_O_2_ (7.9 mmol), CH_3_CN (10 mL), 50 °C and Ar atmosphere.

**Figure 7:**
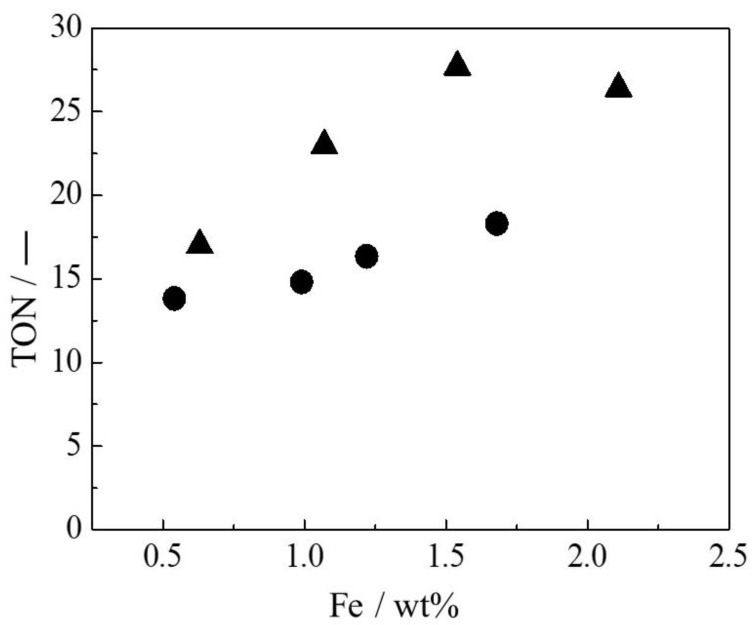
Catalytic activity of Fe(X)L-MYZ-1.0 (▲) and Fe(X)L-YZ (●) catalysts for oxidation of benzene with H_2_O_2_ for 24 h. Reaction condition: Fe in catalysts (7.9 μmol), benzene (7.9 mmol), 30% aqueous H_2_O_2_ (7.9 mmol), CH_3_CN (10 mL), 50 °C, 24 h and Ar atmosphere.

**Figure 8:**
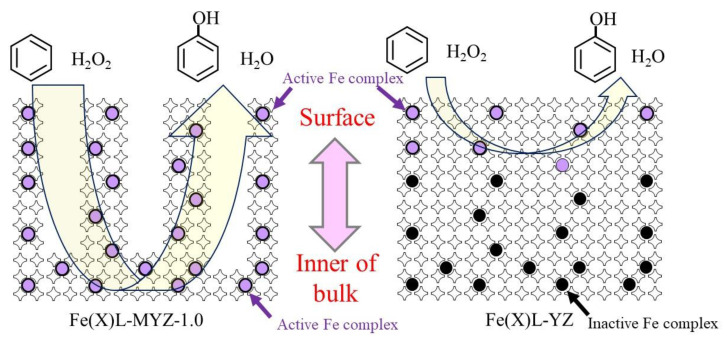
Schematic image of Fe(X)L-MYZ-1.0 and Fe(X)L-YZ catalysts for oxidation of benzene with H_2_O_2_ to phenol.

The partial oxidation of benzene with H_2_O_2_ using Fe(X)L-MYZ-1.0 and Fe(X)L-YZ catalysts proceeded, and phenol was produced selectively on all catalysts. Figure 6 shows the time course for the oxidation of benzene with H_2_O_2_ using Fe(X)L-MYZ-1.0 and Fe(X)L-YZ catalysts. The phenol yield for all Fe complex-containing catalysts increased with increasing time. The initial catalytic activities of the Fe(X)L-MYZ-1.0 catalysts for the oxidation of benzene were higher than those of the Fe(X)L-YZ ones. Figure 7 shows the catalytic activity (TON) for the oxidation of benzene with H_2_O_2_ using Fe(X)L-MYZ-1.0 and Fe(X)L-YZ catalysts for 24 h, as a function of the Fe content in the catalyst. The oxidation activity of benzene increased with increasing X for Fe(X)L-MYZ-1.0 and Fe(X)L-YZ catalysts. The increase in TON with increasing Fe complex content in the catalyst was larger for the Fe(X)L-MYZ-1.0 catalyst than for the Fe(X)L-YZ catalyst. Hence, the Fe(X)L-MYZ-1.0 catalyst has higher catalytic activity per Fe complex when the Fe complex content is increased. This improvement in catalytic activity may be due to the enhancement in the diffusion rate of both benzene and H_2_O_2_ substrates in MYZ-1.0. In particular, in the case of Fe(X)L-MYZ-1.0, the [Fe(terpy)_2_]^2+^ complexes near the surface and in the bulk of MYZ could act as active sites for the oxidation of benzene (Figure 8).

**Figure 9:**
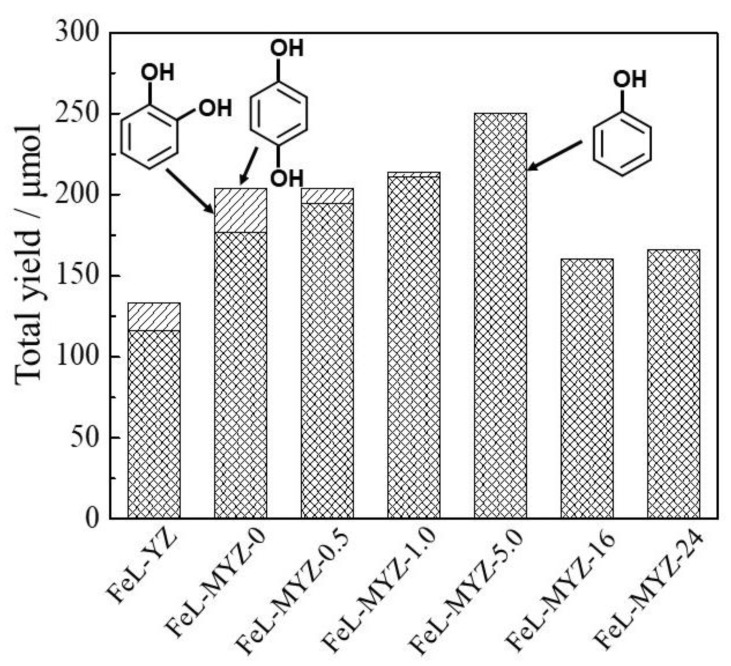
Catalytic activity of FeL-MYZ-t and FeL-YZ catalysts for oxidation of benzene with H_2_O_2_. Reaction condition: Fe in catalysts (16 μmol), benzene (7.9 mmol), 30% aqueous H_2_O_2_ (7.9 mmol), CH_3_CN (5 mL), 50 °C, 24 h and Ar atmosphere.

The partial oxidation of benzene with H_2_O_2_ using the FeL-MYZ-t and FeL-YZ catalysts with the same Fe content (0.9–1.2 wt. %) in Table S1 proceeded. Phenol was produced as the main product, while o-catechol and traces of hydroquinone were produced as by-products (Figure 9). The catalytic activities of the FeL-MYZ-t catalysts were higher than those of the FeL-YZ catalyst. The catalytic activity of FeL-MYZ-t increased to t = 5.0 and decreased with further increases in t. The phenol selectivity of FeL-MYZ-t increased with increasing t. These results suggest that the catalytic activity of FeL-MYZ-t and phenol selectivity increased with the increasing mesopore size of the zeolite support (average pore diameter (d_p_), mesopore volume (V_meso_), and surface area (S_meso_) in Table 1 and pore distribution in Figure 2), supporting the hypothesis shown in Figure 8. The FeL-MYZ-5.0 catalyst exhibited the best catalytic activity for the oxidation of benzene, with H_2_O_2_ to phenol among the catalysts tested.

## 3. Materials and Methods

### 3.1. General

Na ion-exchanged Y-type zeolite (Na-Y) with SiO_2_/Al_2_O_3_ = 5.5 was supplied by Tosoh Co. The following chemicals were used as received: FeSO_4_∙7H_2_O (Wako, > 99%), sodium nitrate (Wako, 99.0%), ethylenediaminetetraacetate acid (TCI, 98.0%), ethylenediaminetetraacetate acid disodium salt (Wako, 99.5%), sodium hydroxide (Wako, 93%), 2,2′;6′,2″-terpyridine (TCI, 98.0%), methanol (Wako, 99.8%), 30% aqueous hydrogen peroxide (Wako, 30–35.5%), benzene (Wako, 99.5%), phenol (Wako, 99.0%), catechol (Wako, 99.0%), hydroquinone (Wako, 99.0%), *o*-dichlorobenzene (Wako, 98.0%), and acetonitrile (Wako, 99.5%).

ICP-AES and CHN elemental analyses of all catalysts were carried out after the sample was dissolved into a HF solution. The powder XRD patterns of the catalysts were collected on a Rigaku MiniFlex II diffractometer using CuK*α* radiation. The Brunauer–Emmet–Teller (BET) surface area measurements were conducted to determine the specific surface areas and pore diameters of the samples by performing N_2_ adsorption experiments at 77 K using a BEL Japan Bellsorp-max instrument. The UV-vis spectra were recorded on a Hitachi U-4000 spectrometer for solid samples. Gas chromatography (GC, Shimadzu GC-2014) was performed using a flame ionization detector equipped with a DB-1MS capillary column (internal diameter = 0.25 mm and length = 30 m) at the nature of the non-polar liquid phase.

### 3.2. Preparation of Mesoporous Y-Type Zeolite

The mesoporous Y-type zeolites were prepared by the sequential processes of acid (H_4_EDTA), base (NaOH), and acid (Na_2_H_2_EDTA) treatments of commercial Y-type zeolite (Na-Y, YZ) as follows.

A suspension of YZ (6.7 g), ethylenediaminetetraacetate acid (3.2 g), and water (100 mL) was stirred at 100 °C for 6 h followed by drying at room temperature under vacuum after filtration. A suspension of the obtained solid, sodium hydroxide (0.8 g), and water (100 mL) was stirred at 65 °C for a set time (t = 0–24 h), followed by filtration and drying at room temperature under vacuum. A suspension of the obtained solid, ethylenediaminetetraacetate acid disodium salt (4.1 g) and water (100 mL) was stirred at 100 °C for 6 h followed by filtration and drying at room temperature under vacuum to obtain a white powder. The white powder was called the acid and base-treated zeolite (MYZ-t).

### 3.3. Preparation of the Fe Complexes Encapsulated into Zeolite

Among MYZ-t, MYZ-1.0, with the highest mesopore volume, was adopted as a typical mesoporous Y-type zeolite. YZ and MYZ-1.0 (2.5 g) were ion-exchanged by a conventional method using an aqueous solution (150 mL) of FeSO_4_∙7H_2_O (arbitrary amount) to yield iron(II) ion-exchanged Y-type zeolite (Fe(X)-YZ and Fe(X)-MYZ-1.0). The X calculated from ICP-AES represents the weight percentage concentration (wt. %) of Fe in the sample. Fe-MYZ-t (t = 0–24) with the same Fe content (0.9–1.2 wt. %) was also prepared by the ion exchange of MYZ-t (2.0 g) with FeSO_4_∙7H_2_O (0.083 g). The prepared Fe(X)-YZ, Fe(X)-MYZ-1.0, and Fe-MYZ-t (1.0 g) were heated under reflux in an aqueous solution (100 mL) of 2,2′;6′,2″-terpyridine (terpy) (arbitrary amount) for 20 h, followed by filtration, washing with water and methanol using a Soxhlet extractor, and drying at room temperature under vacuum to afford Fe(X)L-YZ, Fe(X)L-MYZ-1.0, and FeL-MYZ-t as a purple powder.

### 3.4. Catalytic Oxidation of Benzene

The catalytic oxidation of benzene was carried out in a glass tube reactor. A typical procedure was as follows: catalyst (Fe: 7.9 or 16 μmol), MeCN solvent (5 or 10 mL), and benzene (7.9 mmol) were charged, and 30% aqueous hydrogen peroxide (7.9 mmol) was added to a glass tube reactor under an Ar atmosphere. The reaction was carried out at 50 °C. After the reaction, triphenylphosphine as a quencher and o-dichlorobenzene as an internal standard was added to a glass tube reactor. The products were identified by comparing the peak intensity and retention time for GC-FID with authentic samples. The turnover number (TON) is defined as the total yield [mol] per amount of Fe [mol] contained in the catalyst.

## 4. Conclusions

Mesoporous Y-type zeolite (MYZ) was prepared by the acid–base treatment of commercial Y-type zeolite (YZ) at various base treatment times. The mesopore volume was a maximum when the base treatment time was 1.0 h. MYZ had six times higher mesopore volume than YZ. The mesoporous zeolite catalyst (Fe(X)L-MYZ) with the iron complex encapsulated in mesoporous zeolite was characterized. Fe(X)L-MYZ and Fe(X)L-YZ catalysts with different iron complex contents were used for benzene oxidation, with H_2_O_2_ as the oxidant. Phenol was selectively obtained with all catalysts. For the same amount of iron complex, Fe(X)L-MYZ catalyst had higher catalytic activity than Fe(X)L-YZ catalyst. For both catalysts, the catalytic activity increased with increasing iron complex content. The effect of the iron complex content was greater for the Fe(X)L-MYZ catalyst than for the Fe(X)L-YZ catalyst.

## Figures and Tables

**Table 1:** N_2_ absorption–desorption parameters of MYZ-t and YZ at 77 K.

	S_BET_^(a)^ /m^2^ g^−1^	V_m_^(b)^ /cm^3^ g^−1^	V_total_^(c)^ /cm^3^ g^−1^	d_p_^(d)^ /nm	V_meso_^(e)^ /cm^3^ g^−1^	V_micro_^(f)^ /cm^3^ g^−1^	S_meso_ ^(g)^ /m^2^ g^−1^	S_micro_ ^(h)^ /m^2^ g^−1^	S_total_ ^(i)^ /m^2^ g^−1^
YZ	960	221	0.61	2.5	0.11	0.54	17	1745	1755
MYZ-0	828	190	0.77	3.7	0.43	0.56	354	1257	1376
MYZ-0.5	822	189	1.09	5.3	0.65	0.39	245	1344	1666
MYZ-1.0	775	178	1.10	5.7	0.73	0.33	270	1177	1522
MYZ-5.0	637	146	0.88	5.5	0.55	0.30	206	1031	1291
MYZ-16	631	145	0.85	5.4	0.51	0.30	194	1049	1292
MYZ-24	639	147	0.79	4.9	0.45	0.36	168	1147	1313

(a) BET specific surface area. (b) Monomolecular adsorption volume. (c) Total pore volume. (d) Average pore diameter. (e) Mesopore volume. (f) Micropore volume. (g) Mesopore specific surface area. (h) Micropore specific surface area. (i) Total specific surface area.

## Supplementary Materials

The following supporting information can be downloaded at: https://www.mdpi.com/article/10.3390/molecules27206852/s1, Table S1: ICP-AES results for FeL-MYZ-t and FeL-YZ catalysts. Figure S1: N2 absorption–desorption isotherm of MYZ-t and YZ at 77 K. Figure S2: XRD patterns of Fe(X)-YZ. Figure S3: XRD patterns of Fe(X)L-YZ catalysts. Figure S4: XRD patterns of Fe-MYZ-t and Fe-YZ. Figure S5: XRD patterns of FeL-MYZ-t and FeL-YZ catalysts. Figure S6: UV-vis. spectra of Fe(X)L-YZ catalysts. Figure S7: UV-vis. spectra of FeL-MYZ-t and FeL-YZ catalysts.
