# Supplementary material for: Direct Hydroxylation of Benzene with Hydrogen Peroxide Using Fe Complexes Encapsulated into Mesoporous Y-Type Zeolite
Source: Molecules. 2022 Oct 13;27(20):6852. doi: 10.3390/molecules27206852 (PMC9612136; doi:10.3390/molecules27206852)
Supplement: Supplementary file 1 [file molecules-27-06852-s001.zip › molecules-1922763-supplementary.pdf]

Direct hydroxylation of benzene with hydrogen peroxide using Fe complexes encapsulated into mesoporous Y-type zeolite

Syuhei Yamaguchi,* Yuito Ishida, Hitomu Koga, and Hidenori Yahiro

Department of Materials Science and Biotechnology, Graduate School of Science and Engineering, Ehime University, 3 Bunkyo-cho, Matsuyama 791-8577, Japan

*Corresponding author: Tel: +81-89-927-9927.

E-mail: syuhei@ehime-u.ac.jp (S. Yamaguchi)

Table S1. ICP-AES results for FeL-MYZ-t and FeL-YZ catalysts.

	Si/ wt.%	Al/ wt.%	Na/ wt.%	Fe/ wt.%	Si/Al
FeL-YZ	25.9	7.9	6.1	0.9	3.1
FeL-MYZ-0	28.1	4.4	2.7	1.1	6.1
FeL-MYZ-0.5	26.2	6.5	4.8	1.0	3.9
FeL-MYZ-1.0	26.1	6.7	4.7	1.2	3.7
FeL-MYZ-5.0	26.6	7.2	5.0	1.0	3.5
FeL-MYZ-16	26.0	7.5	5.1	1.2	3.3
FeL-MYZ-24	26.0	7.4	5.2	1.2	3.4

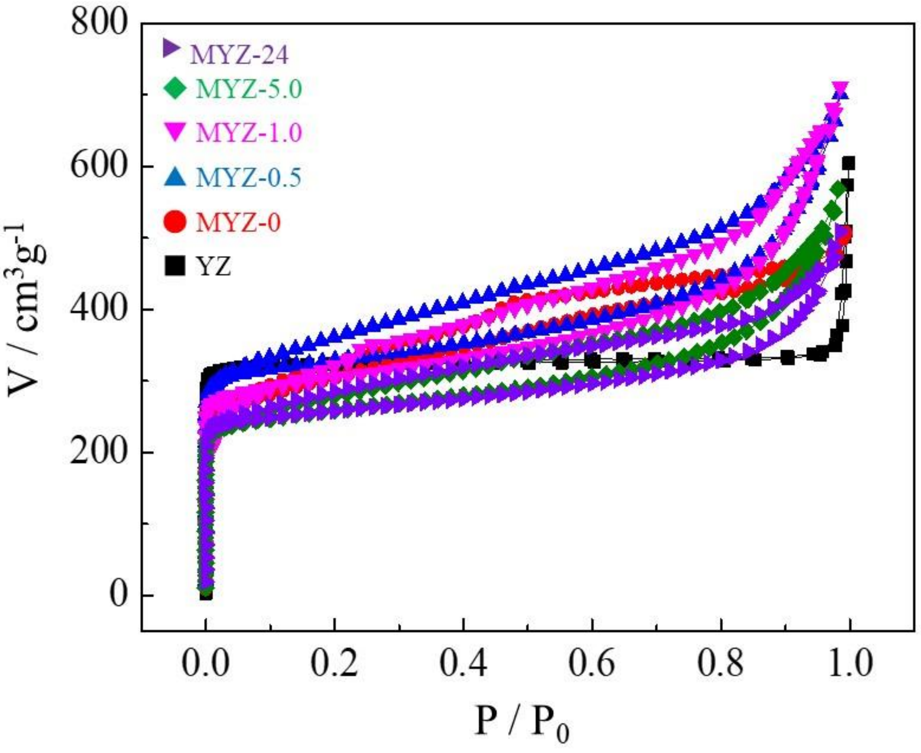

Figure S1. N_2 absorption-desorption isotherm of MYZ-t and YZ at 77 K.

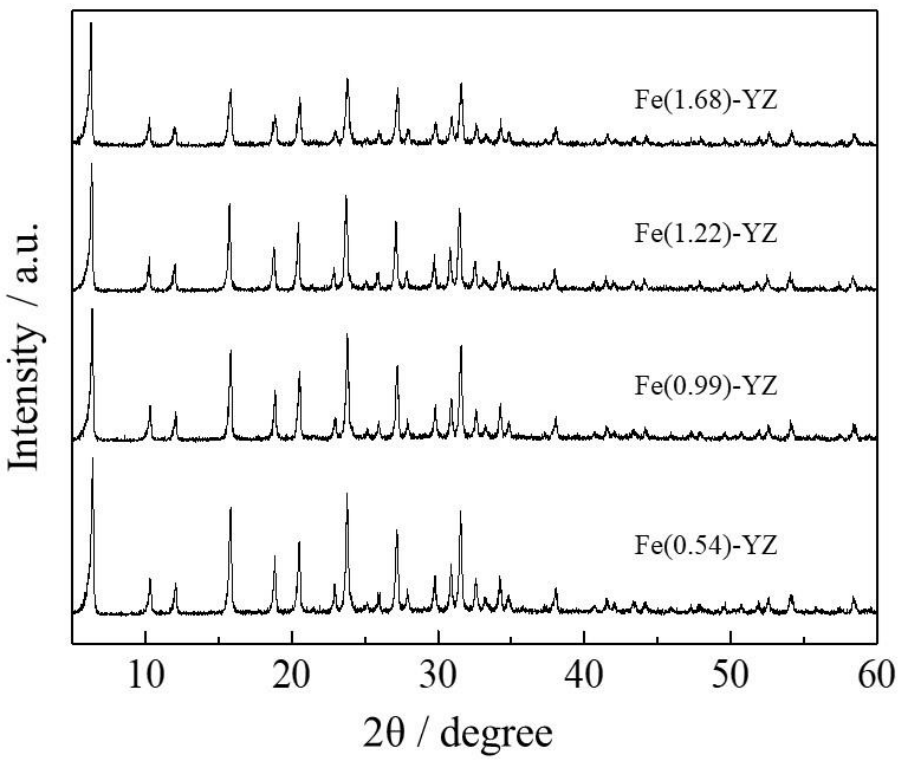

Figure S2. XRD patterns of Fe(X)-YZ.

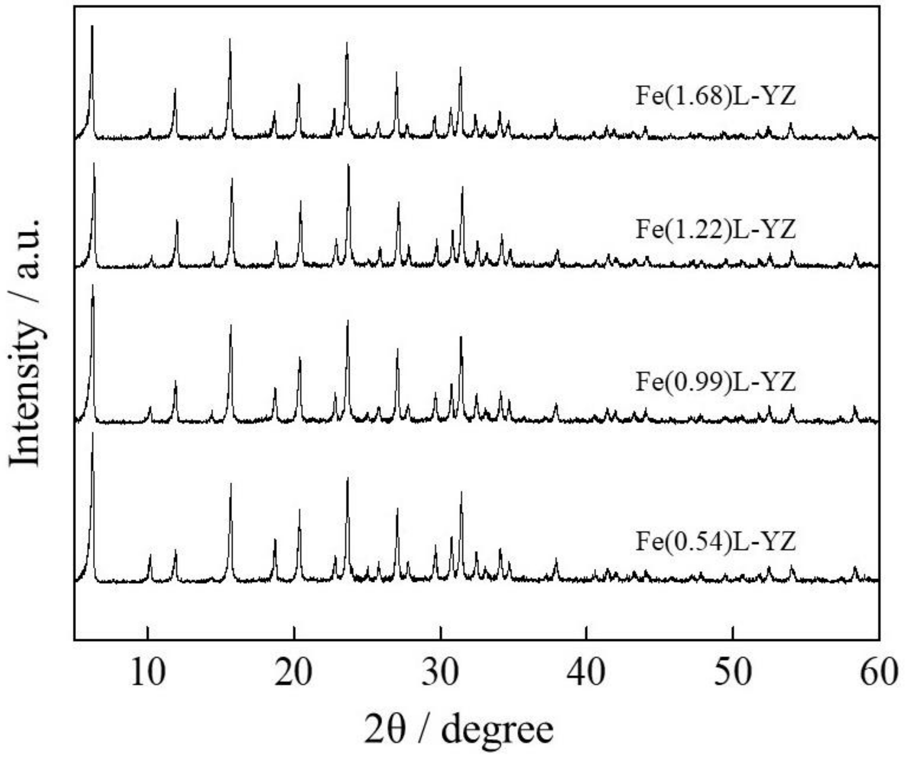

Figure S3. XRD patterns of Fe(X)L-YZ catalysts.

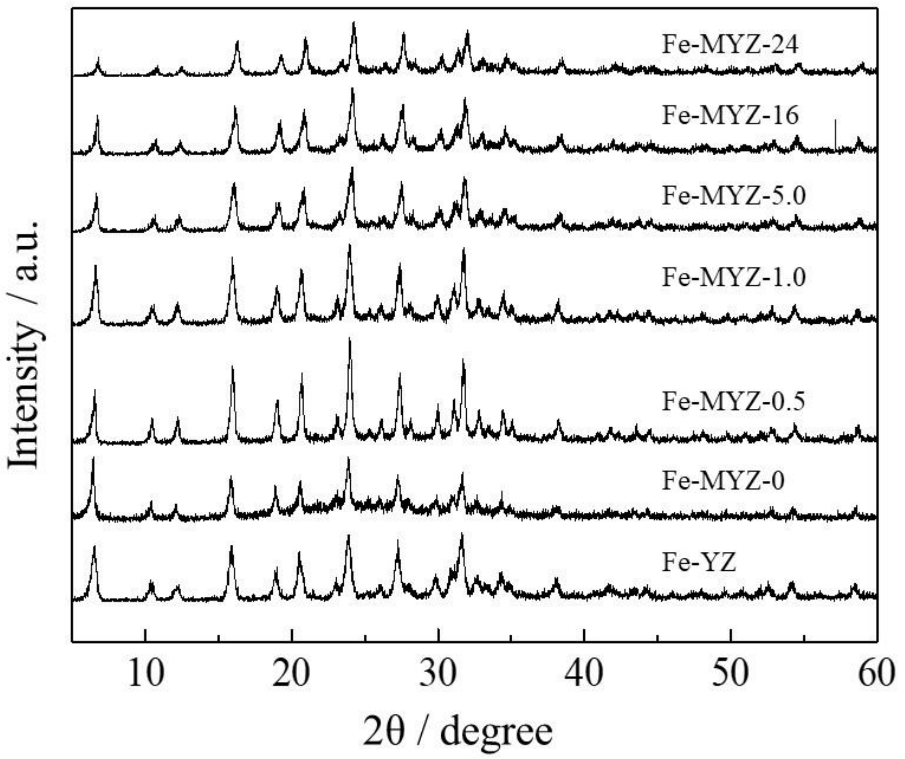

Figure S4. XRD patterns of Fe-MYZ-t and Fe-YZ.

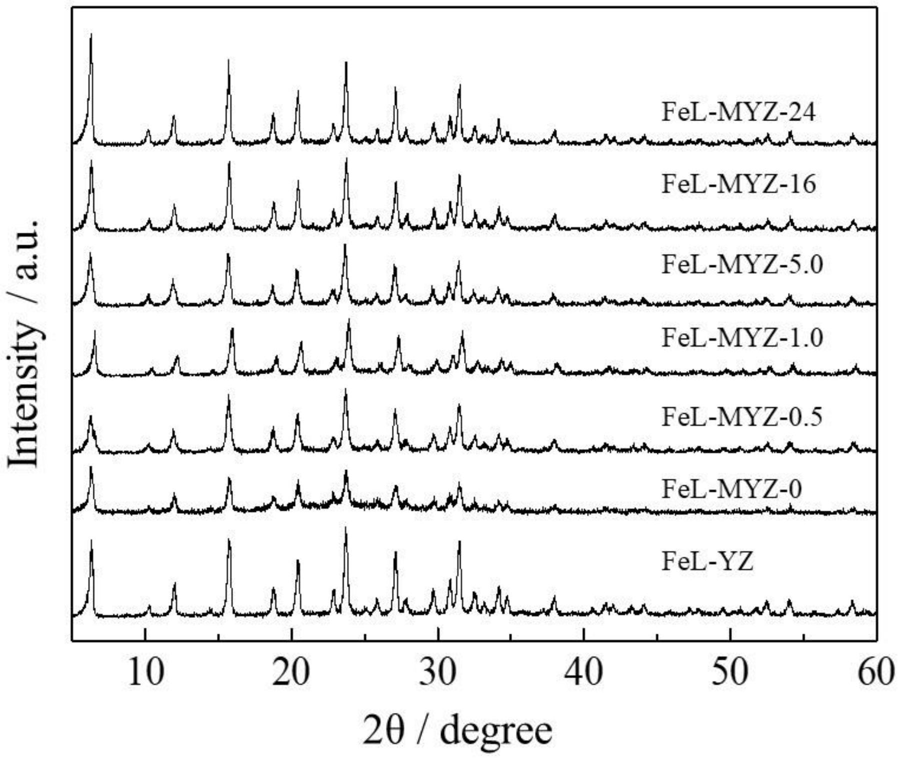

Figure S5. XRD patterns of FeL-MYZ-t and FeL-YZ catalysts.

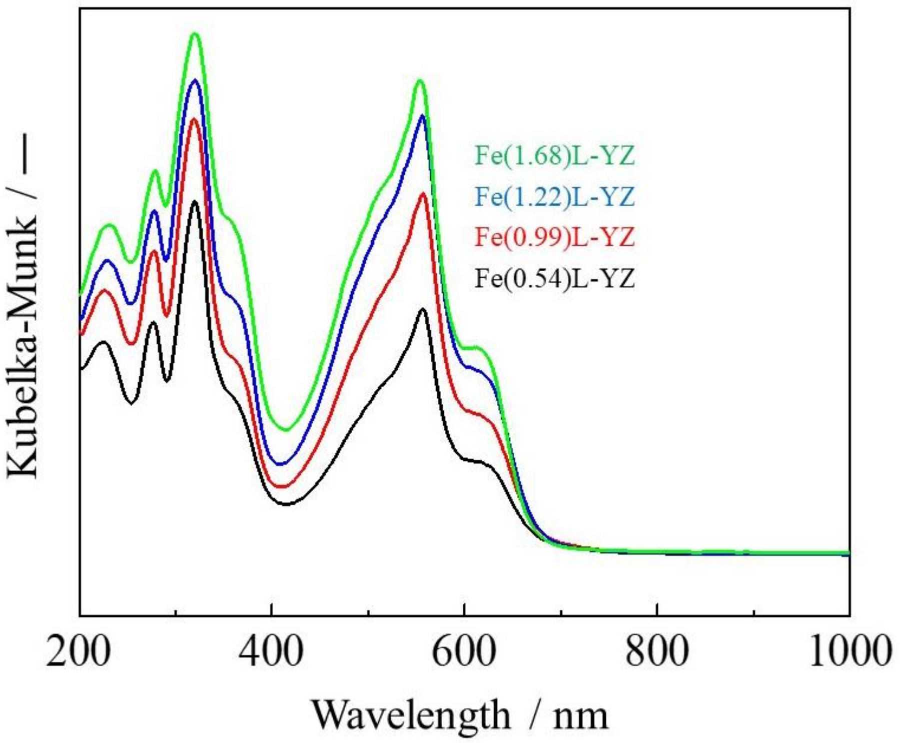

Figure S6. UV-vis. spectra of Fe(X)L-YZ catalysts.

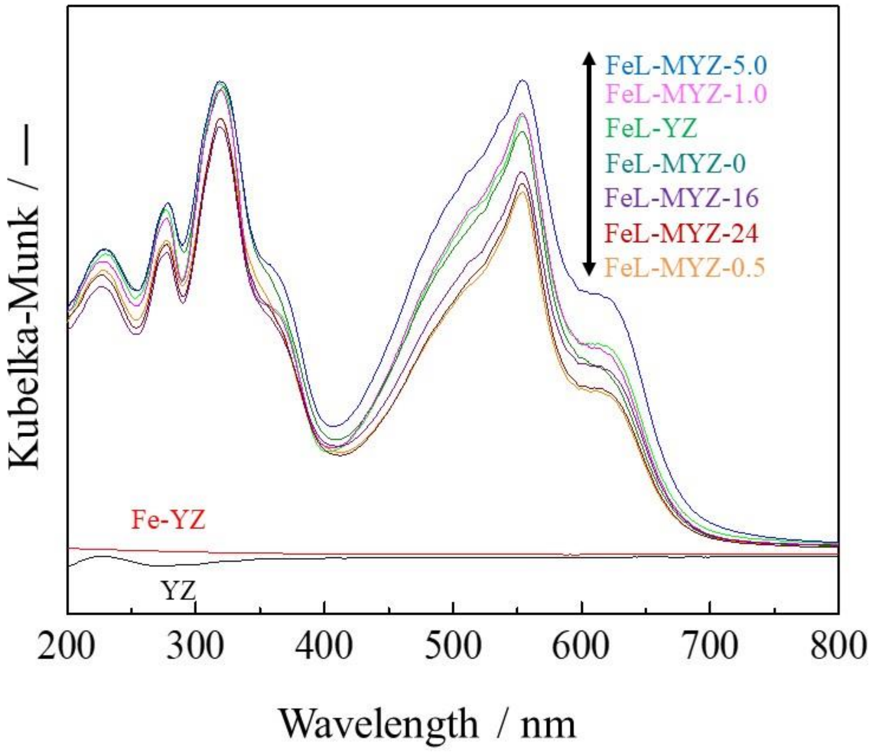

Figure S7. UV-vis. spectra of FeL-MYZ-t and FeL-YZ catalysts.
